# Patient perspectives on tooth replacement: A cross-sectional survey

**DOI:** 10.6026/973206300211310

**Published:** 2025-06-30

**Authors:** Sai Krishna Athota, K. Varun Reddy, Harikrishna Modalavalasa, Bhargavi Katta, Ravi Shankar Yalavarthy, Prachi Krishna Kant Pandey

**Affiliations:** 1 Clinician, Windsormill, MD, USA; 2 Sree Sai Dental College and Research Institute, Srikakulam, Andhra Pradesh, India; 3 Department of Prosthodontics, GITAM Dental College and Hospital, Visakhapatnam, India; 4 Department of Prosthodontics, Drs. S and NR SIDS, Gannavaram, India; 5 Mamata Dental College, Khammam, India; 6 Clinician, Mahavir Arogya Sansthan, Patna, Bihar, India

**Keywords:** Awareness, missing teeth, patient attitudes, dental prosthesis, cross-sectional survey

## Abstract

Awareness, attitudes, knowledge and preferences regarding tooth replacement among 200 adults aged 30-70 years using a validated 26-item questionnaire is of interest. Results showed that 66% of participants had a positive attitude towards treatment and 5% of participants showed negative awareness, while 38% demonstrated good awareness and 29% had poor understanding of available options. Socio-demographic factors are including age, education, affordability, influenced decisions and expectations. The findings highlight the need for targeted awareness programs to improve knowledge and encourage early adoption of dental prosthetic care in adult population.

## Background:

Teeth are an important component of the oral cavity and solely work as a functional component for mastication, phonation, esthetic component for an individual's appearance and aids in the digestive process, hence forming a healthy stomatognathic system [1]. Structurally, teeth consist of enamel, dentin and pulp, each contributing to their durability and vitality. Enamel protects against external forces and bacterial invasion, while dentin provides strength and flexibility. The pulp houses nerves and blood vessels essential for tooth function and regeneration. Beyond their physical attributes, teeth play a pivotal role in digestion by enabling proper food breakdown, which aids in nutrient absorption and promotes general health. The importance of teeth extends to communication and self-esteem. Teeth facilitate clear speech by forming sounds critical to effective communication. A healthy set of teeth also supports facial structure, prevents sagging and maintains aesthetic harmony. Moreover, teeth significantly impact confidence; their appearance influences social interactions and mental well-being. Partial or complete edentulism can be caused due to various reasons and affect a person psychologically, functionally and socially [2]. Posterior teeth act as a functional unit for mastication and anterior teeth for esthetics; hence missing teeth leads to a loss of function, affecting the physiological health and later affecting the psychological and social behaviour of an individual. Therefore, losing teeth reduces a person's quality of life psychologically, socially and emotionally. The oral-systemic connection highlights the broader implications of oral health. Poor oral hygiene can exacerbate systemic conditions such as cardiovascular disease, diabetes, respiratory issues and adverse pregnancy outcomes. Gum diseases like periodontitis allow harmful bacteria to enter the bloodstream, triggering inflammation that impacts overall health. Addressing oral health is thus critical for preventing systemic complications and enhancing general well-being. There are various treatment modalities available for the replacement of missing teeth, namely, removable partial and complete dentures, fixed dental prosthesis and implant-supported prosthesis [3]. Most of the patients feel that the fixed prosthesis gives a better feeling in the mouth and appears more natural. Also, fixed prosthesis is esthetically more attractive than removable prosthesis and less annoying in the mouth. The selection of treatment options depends on the patient's oral condition, economic condition, acceptance and awareness of the treatment plan [4]. Advancements in dental technology have revolutionized patient care. Innovations such as digital impressions, 3D imaging, AI-powered diagnostics and immediate-load implants enable precise treatment planning and enhance patient satisfaction. Emerging fields like regenerative dentistry using stem cell therapy or tissue engineering, promise future breakthroughs in tooth replacement by regenerating natural structures. These days, social media and technology has increased people's awareness leading to a growth of specific needs in patients about the treatment and procedures. At times, it becomes extremely difficult for dentists to meet the needs of their patients [5]. Therefore, it is of interest to assess awareness, attitudes, needs and preferences regarding tooth replacement among the adult population.

## Materials and Methods:

## Methodology:

## Study design:

A Cross-Sectional survey was conducted among 200 patients visiting a dental clinic in one year. The Questionnaire mainly consisted of 15 questions divided into three sections.

The study utilized a structured, validated 26-item questionnaire divided into three sections:

[1] Demographic Data (5 items): Age, gender, education level, occupation and geographic region.

[2] Clinical and Treatment Preferences (5 items): Number of missing teeth, prosthetic status (using WHO criteria), treatment options, affordability and time since tooth loss.

[3] Awareness, Attitudes and Needs (16 items): Knowledge of replacement options, consequences of tooth loss, sources of information, barriers to treatment and preferences for aesthetics/functionality.

The questionnaire was validated by a panel of dental professionals and pre-tested for clarity and relevance.

## Study population:

## Inclusion criteria:

[1] Patients aged 30-70 years.

[2] New patients visiting the clinic during the study period.

[3] Willingness to provide informed consent.

## Exclusion criteria:

[1] Patients with cognitive, physical or communication impairments.

[2] Uncooperative individuals or those unwilling to participate.

[3] Patients under 30 or over 70 years of age.

## Data collection protocol:

[1] Participant recruitment: Eligible patients were briefed on the study's objectives and written consent was obtained.

[2] Clinical examination: Intraoral assessments were performed using the WHO Oral Health Assessment Form to document.

[3] Prosthetic Status: Categorized as no prosthesis (Code 0), bridge(s) (1-2), partial denture (3), combined prostheses (4), or full denture (5).

[4] Prosthetic need: Classified as no need (0), single/multi-unit prosthesis (1-3), full prosthesis (4), or not recorded (9).

[5] Questionnaire administration: The self-administered questionnaire was distributed in English. For participants with limited literacy, trained staff provided verbal assistance.

## Questionnaire structure:

The questionnaire was prepared in English language and given to the patients. In the first part of the questionnaire, basic demographic data questions such as name, sex, age, region, country were collected. The second part included questions regarding various treatment options for the patients for replacing the missing teeth such as number of teeth to be replaced, type of treatment required (removable or fixed). The third part included questions regarding the patient having knowledge about replacement of missing tooth, various types of replacement options available, awareness of consequences of not replacing missing tooth/teeth, demands and needs of the patient for the replacement of missing tooth whether it was for aesthetics or function or both aesthetic and function. After intra oral examination was performed in the dental clinic, following findings were filled by the examiners in the questionnaire with the help of World Health Organization (WHO) oral health assessment form.

## The instrument comprised three sections:

Demographics: Name, captured age, gender, education, occupation and residence.

## Clinical Data:

[1] Tooth loss history (duration, number, location: anterior/posterior).

[2] Applicable treatments (removable/fixed dentures, implants).

[3] Affordability assessments (yes/no).

## Awareness and Preferences:

[1] Knowledge of treatment options (implants, bridges, dentures).

[2] Information sources (dentists, social media and peers).

[3] Barriers to care (cost, fear, lack of awareness).

[4] Priorities (aesthetics vs. functionality).

## The WHO oral health assessment form includes:

## Prosthetic status:

[1] Code 0: No prosthesis

[2] Code 1: Bridge

[3] Code 2: More than one bridge

[4] Code 3: Partial denture

[5] Code 4: Both bridge(s) and partial denture(s)

[6] Code 5: Full removable denture

[7] Code 9: Not recorded.

## Prosthetic need:

[1] Code 0: No prosthesis needed

[2] Code 1: Need for one-unit prosthesis

[3] Code 2: Need for multi-unit prosthesis

[4] Code 3: Need for a combination of one and/or multi-unit prosthesis

[5] Code 4: Need for full prosthesis (replacement of all teeth)

[6] Code 9: Not recorded.

The collected data were assessed, tabulated and the obtained results were statistically analysed.

## Statistical analysis:

[1] Descriptive Statistics: Frequencies and percentages summarized categorical variables (*e.g.*, prosthetic needs, awareness levels).

[2] Inferential Statistics:

a. Chi-square or Fisher's exact tests compared associations between sociodemographic factors (age, education) and treatment preferences.

b. Logistic regression identified predictors of awareness (*e.g.*, education level, internet access).

[3] Software: Analyses were performed using SPSS Statistics v28.0, with significance set at p < 0.05.

Table 1 (see PDF) outlines the WHO prosthetic status and need codes, which are used to classify an individual's current prosthetic condition and their need for prosthetic treatment. A code of "0" indicates no prosthesis, while codes "1" and "3" refer to having a single bridge or partial denture, respectively. For prosthetic needs, "2" denotes a requirement for a multi-unit prosthesis, and "4" indicates the need for a full prosthesis. Table 2 (see PDF) presents examples of items from a questionnaire designed to assess patient perceptions. It includes questions on the importance of natural-looking teeth and barriers to seeking treatment, with response options such as "Very/Somewhat/Not important" and choices like cost, fear, or lack of awareness. This revised section adheres to STROBE guidelines for cross-sectional studies, ensuring methodological transparency and reproducibility.

## Ethical consideration:

Before conducting the survey, a written consent was obtained from all the participants. All the patients were informed regarding the objectives and aims of the study.

## Data analysis:

Qualitative variables were reported as frequencies, absolute values, or percentages. Comparisons between groups were evaluated using the chi-square test or Fisher's exact test, as appropriate. The statistical analysis was done at a p-value of < 0.05 and analysis was performed through SPSS Statistics software.

## Results:

A total of 200 adult patients participated in the survey. Among them, 25% (n = 50) had no formal education and reported limited knowledge regarding dental treatments.

When evaluating treatment attitudes:

[1] 132 participants (66%) exhibited a positive attitude toward replacing missing teeth,

[2] 32 participants (16%) were neutral and

[3] 10 participants (5%) demonstrated a negative attitude, citing concerns related to cost, treatment longevity and fear of procedures.

These findings suggest a predominantly favourable perception of dental rehabilitation among respondents when evaluating treatment attitudes (Figure 1 - see PDF).

Awareness levels varied across the sample:

[1] 38% (n = 76) of participants had better awareness,

[2] 32.3% (n = 65) had some awareness and

[3] 29% (n = 58) were found to have poor awareness of treatment modalities and the consequences of not replacing missing teeth and on the awareness on tooth replacement options (Figure 2 - see PDF).

Participants with higher educational attainment or exposure to dental professionals and digital sources demonstrated better awareness scores. Additionally, younger individuals (<50 years) were more likely to value treatment longevity, particularly favouring implant-based solutions, while older patients prioritized affordability and familiarity with removable prostheses. Table 3 (see PDF) shows the relationship between awareness levels of dental prosthetics and education levels. It demonstrates a clear trend: individuals with higher education tend to have better awareness, while those with no formal education largely fall into the "poor" awareness category. As shown in Table 3 (see PDF) , participants with no formal education were more likely to exhibit poor awareness (n = 30), whereas those with higher education most frequently reported better awareness (n = 41). A positive trend was observed between increasing education level and awareness about the consequences and treatment options for missing teeth (Figure 3 - see PDF).

## Discussion:

This study evaluated the awareness, attitudes and preferences of adults regarding the replacement of missing teeth in adult population. The findings revealed that while a majority demonstrated a positive attitude toward prosthetic rehabilitation, significant knowledge gaps persisted-especially among those with no formal education [1]. Participants with higher education levels and digital exposure were more aware of modern options like implants and bridges, whereas those with limited literacy often lacked understanding of treatment necessity or benefits [2]. Tooth loss commonly leads to a decline in mastication and aesthetic appearance, motivating individuals to seek replacement primarily for functional and visual reasons. However, ignorance about treatment options remains a major deterrent to timely rehabilitation [3, 4]. Following tooth extraction, alveolar bone resorption occurs and adjacent or opposing teeth tend to migrate into the edentulous space. This migration contributes to occlusal disharmony and increases the complexity of future prosthetic treatment [5, 6]. Early replacement of missing teeth plays a crucial role in preserving bone structure, improving speech and chewing efficiency and maintaining facial musculature support. These factors collectively enhance both appearance and self-confidence [7, 8]. As observed in this study, aesthetic expectations and treatment longevity were key concerns among younger adults, while older individuals were more cost-sensitive-a trend consistent with socio-economic barriers highlighted in the literature [9, 10]. The selection of prosthetic modality is influenced by multiple factors including awareness, affordability and the patient's desire for comfort or permanence.

## Commonly offered treatments include:

[1] Removable partial dentures (RPDs) and Complete Dentures (CDs): These are affordable options widely used due to their simplicity in fabrication. However, they are less comfortable and stable compared to fixed alternatives [11, 12].

[2] Fixed partial dentures (FPDs): Permanently cemented prosthesis offering better stability and aesthetics. FPDs are preferred by patients seeking non-removable solutions for one or more missing teeth [13, 14].

[3] Dental implants: Considered the gold standard in modern prosthodontics, implants mimic natural tooth roots, preserve alveolar bone, restore full function and provide high aesthetic value. They are ideal for patients prioritizing durability and appearance but remain inaccessible for many due to cost concerns [15, 16].

Financial concerns and fear of pain were noted as primary barriers to treatment among participants. This reflects the need for enhanced patient education and counselling to bridge knowledge gaps and improve acceptance of dental prostheses [17]. Public health campaigns addressing affordability issues alongside targeted awareness programs could play a pivotal role in improving access to dental care across diverse population.

## Conclusion:

A growing awareness among the adults regarding the importance of tooth replacement, driven in part by increased exposure to digital health content and professional outreach is seen. However, significant knowledge gaps persist, particularly among underserved and less-educated populations.

## Funding statement:

Nil
